# Early recurrence of thoracolumbar intervertebral disc extrusion after surgical decompression: a report of three cases

**DOI:** 10.1186/1751-0147-52-10

**Published:** 2010-02-05

**Authors:** Øyvind Stigen, Nina Ottesen, Karin H Jäderlund

**Affiliations:** 1 Department of Companion Animal Clinical Sciences, Norwegian School of Veterinary Science, PO Box 8146, 0033 Oslo, Norway

## Abstract

Thoracolumbar disc extrusions were diagnosed in three chondrodystrophic dogs with paraparesis of up to three days duration. All cases were managed by hemilaminectomy and removal of extruded disc material. In one dog, fenestration of the herniated disc space was also performed. Initially neurological function improved or was unchanged, but from two to ten days postoperatively clinical signs of deterioration became apparent. In all the dogs, recurrence of disc extrusion at the same location as the initial extrusion was diagnosed by computer tomography and at a second surgery abundant disc material was found at the hemilaminectomy site between the dura and an implanted graft of autogenous fat.

## Background

Dogs with thoracolumbar intervertebral disc disease causing severe neurological deficits are commonly treated by surgical decompression of the spinal cord. Decompression is usually obtained by hemilaminectomy with removal of extruded disc material from the spinal canal. Fenestration of intervertebral discs at the time of spinal cord decompression is often performed as an additional procedure to prevent future extrusion of disc material.

Fenestration of the herniated disc at the time of surgical decompression has been recommended to prevent continued extrusion at the same level postoperatively [1,2]. However, arguments against fenestration at the time of decompression are that there is less nucleus material remaining within such a herniated disc and that there is a reduced possibility for more extruded disc material to cause significant cord compression after hemilaminectomy. Prophylactic fenestration can also provoke disc extrusion at adjacent, nonfenestrated disc spaces [2]. Nevertheless, in a prospective MRI study that included 19 chondrodystrophic dogs with a first episode of thoracolumbar disc disease, Forterre *et al. *[3] found that fenestration of the affected disc space did prevent further extrusion of disc material with a subsequent reduction of postoperative complications.

In the above-mentioned study by Forterre *et al. *[3], early (within six weeks) recurrence of disc extrusion was reported in six out of ten dogs with thoracolumbar disc disease that had hemilaminectomy without fenestration. Out of these six, three remained without clinical signs, two developed signs of temporary back pain and one had a deterioration of neurologic status three days after surgery. To the authors' knowledge, no other studies have shown that recurrence of disc extrusion of the same disc causes early postoperative complications in dogs.

This case report presents three chondrodystrophic dogs with recurrence of thoracolumbar disc disease within ten days after hemilaminectomy. All recurrences were at the same disc space as the first extrusion, led to aggravation of paraparesis, were identified by computer tomography (CT) and were confirmed by a second surgery.

## Case presentations

### Dog 1

**Figure 1:**
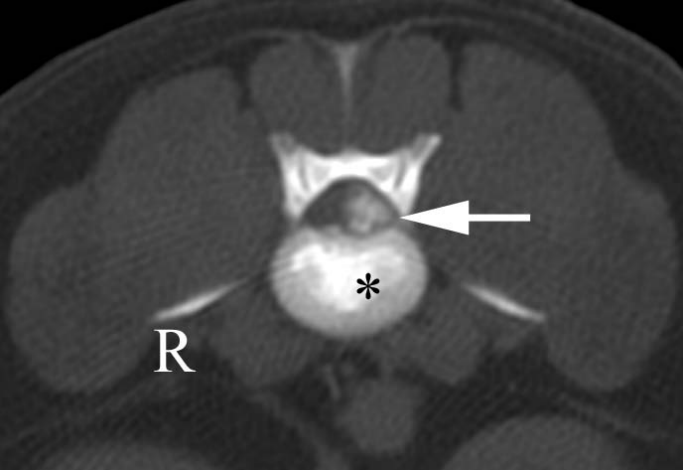
**CT scan in bone window through L2-3 showing calcified material (arrow) to the left in the spinal canal**. The intervertebral disc is calcified (*). These findings suggest extruded disc material causing a severe lateral compression of the spinal cord that was confirmed at surgery.

A six-year-old male wirehaired dwarf dachshund was presented for examination of progressive pelvic limb weakness and incoordination of three days' duration. Intermittent back pain was also reported. Examination revealed kyphosis, severe ambulatory paraparesis and pelvic limb ataxia. Postural reactions were absent in both pelvic limbs. Spinal reflexes and deep pain perception were normal. The symptoms indicated a lesion localized between the T3 and L3 spinal cord segments. Plain radiographs of the thoracolumbar spine showed calcification at the intervertebral disc spaces T12-13, L2-3 and L4-5. CT revealed a disc extrusion lateralised to the left, causing severe spinal cord compression at L2-3 (Figure 1).

A left hemilaminectomy was performed at L2-3 using a high-speed spinal bur. Extruded intervertebral disc material was identified and removed to expose the dura covering the spinal cord. Prior to closure, the hemilaminectomy site was covered by a 3-4 mm thick layer of subcutaneous fat.

**Figure 2:**
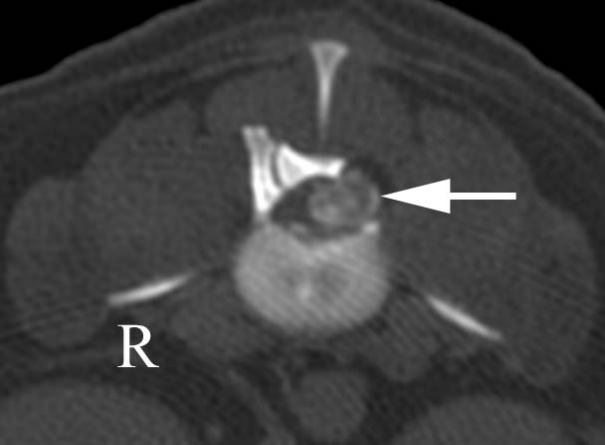
**CT scan in bone window through L2-3 three weeks after left sided hemilaminectomy**. The scan reveals calcified material (arrow) in the spinal canal compressing the spinal cord laterally towards the right. The calcified material was presumed to represent a recurrent disc extrusion that was confirmed at surgery.

For the first seven days following surgery, neurological function gradually improved. Neurological function was unchanged for the next three days before signs of deterioration appeared. Three weeks following surgery, the dog was paraplegic without deep pain perception. Signs of back pain were not observed. CT was repeated and again revealed calcified disc material to the left of a severely compressed spinal cord at L2-3 (Figure 2). A second surgery was performed and abundant disc material was found at the hemilaminectomy site between the dura and the fat graft. The extruded disc material was removed, a new autogenous fat graft placed at the hemilaminectomy site and the wound closed routinely. The dog was discharged seven days following the second surgery and put into an extensive rehabilitation program. At the last check-up one year later, examination revealed ambulatory paraparesis and moderate pelvic limb ataxia.

### Dog 2

**Figure 3:**
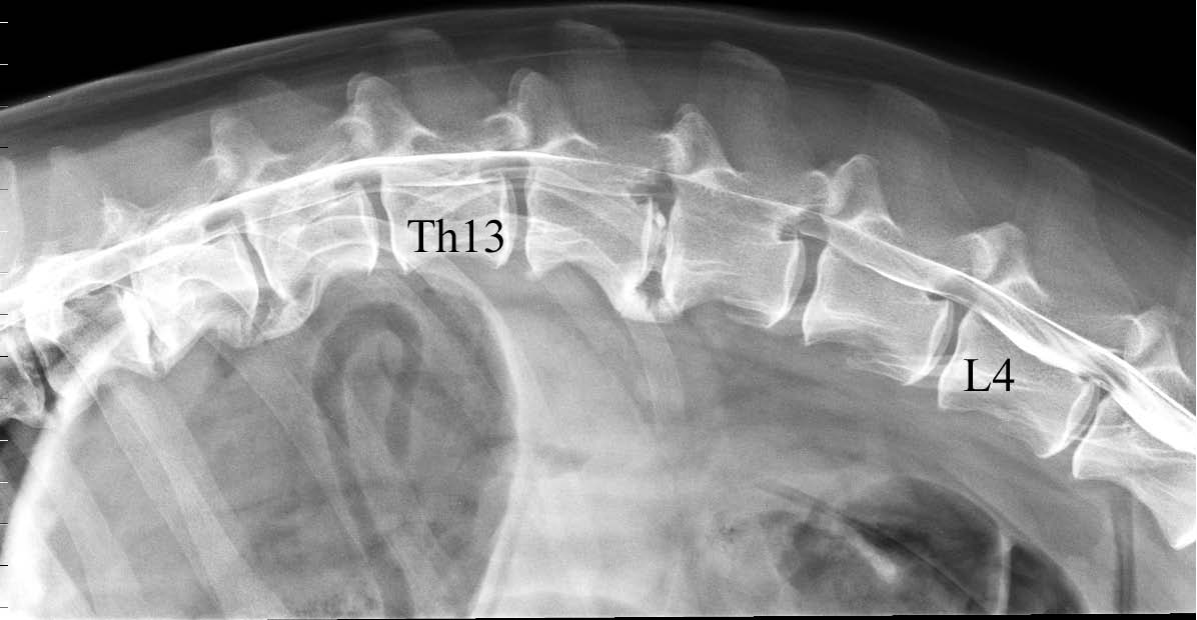
**Lateral myelogram of the thoracolumbal junction**. The L1-2 intervertebral disc is calcified and there is a thinning of the subarachnoid space from L1 to L4. The ventrodorsal myelogram (not included) showed an extradural mass to the right in the spinal canal compressing the spinal cord towards the left from L1 to L3, but most prominent at L1-2.

A nine-year-old neutered male basset hound was presented for examination of paraparesis that had been present for approximately 20 hours. Intermittent back pain and mild pelvic limb ataxia were reported to have occurred over the last year. Examination revealed a non-ambulatory paraparesis. Postural reactions were absent in both pelvic limbs, but spinal reflexes and deep pain perception were normal. A lesion in the T3 to L3 spinal cord segment was presumed. Plain radiographs of the thoracolumbar spine showed spondylosis deformans. There were also calcified intervertebral discs at T10-11 and L1-2. The degree of calcification was evaluated as 'severe' in the T10-11 disc and 'moderate' in the L1-2 disc [4]. A lateral myelogram revealed thinning of the subarachnoid space from L1 to L4 (Figure 3) whereas a ventrodorsal myelogram revealed thinning of the subarachnoid space from T13 to L4. The thinning of the subarachnoid space in both myelograms was caused by an extradural mass. The mass was lateralised to the right and most prominent at L1-2. A diagnosis of disc extrusion at L1-2 causing moderate to severe spinal cord compression at T13 to L3 was made.

A right hemilaminectomy was performed at T13 to L3. A large amount of extruded disc material was found in the extradural space, particularly around the right L1 nerve root. The disc material was removed and an autogenous fat graft placed at the hemilaminectomy site.

The dog was discharged six days following surgery. Neurological function was improved compared with preoperative function. The dog stood without assistance and walked for two to three minutes in a water-walker.

**Figure 4:**
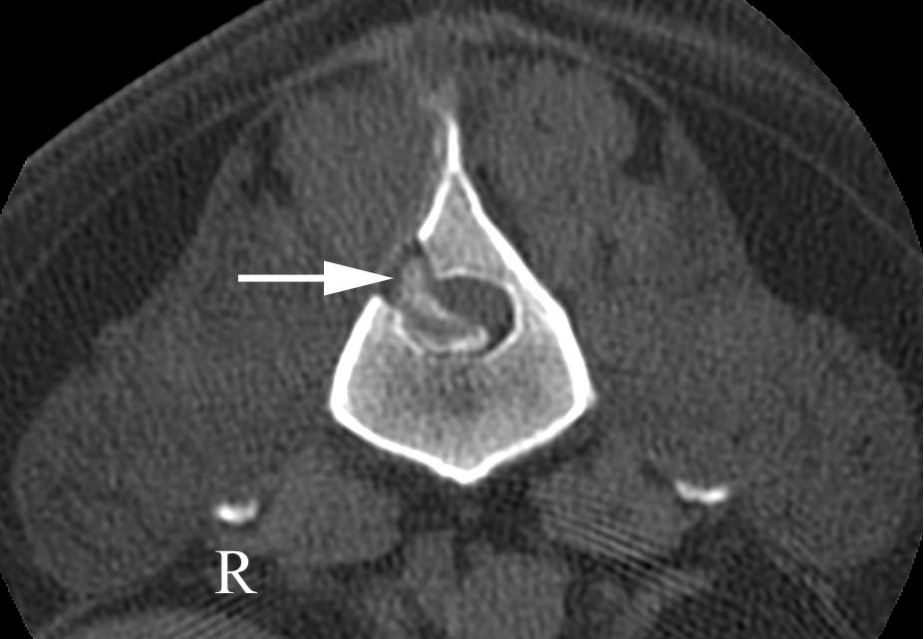
**CT scan in bone window through the center of L2 eleven days after right sided hemilaminectomy**. Calcified material (arrow) in the spinal canal is compressing the spinal cord dorsally and laterally towards the left. These findings suggest a recurrent disc extrusion at L1-2 that was confirmed at surgery.

At suture removal eleven days following surgery, clinical signs of deterioration were obvious. The dog could no longer stand on its pelvic limbs, nor walk in a water-walker. Deep pain perception was decreased in both pelvic limbs. Plain radiographs showed a slight degree of calcification of the L1-2 intervertebral disc. CT revealed calcified material to the right of a compressed spinal cord at L1-2 (Figure 4). A second surgery was performed and abundant disc material was identified beneath the fat graft at the L1-2 site. Extradural disc material was removed and the L1-2 intervertebral disc fenestrated. However, it was only possible to retrieve a small amount of nucleus pulposus during the fenestration procedure. The dog was discharged six days after the second operation. Neurological function was mildly improved compared with preoperative function, but the dog could still not stand on its pelvic limbs. A rehabilitation program including hydrotherapy was commenced. Nine months later good ambulatory abilities, including climbing stairs, were reported.

### Dog 3

**Figure 5:**
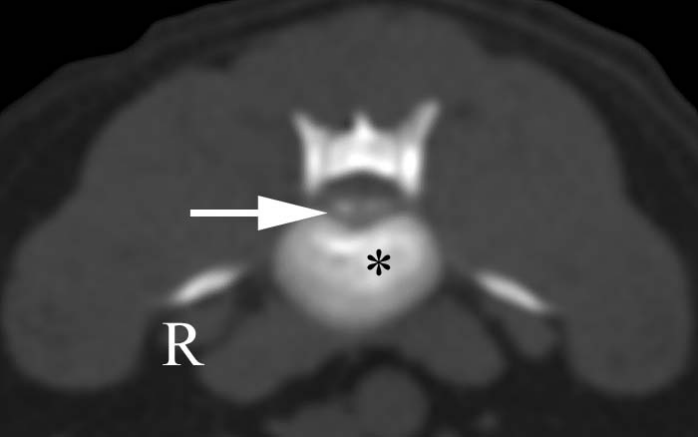
**CT scan in bone window through L3-4 showing calcified material (arrow) ventrally in the spinal canal**. The spinal cord is compressed. The intervertebral disc is calcified (*). These findings suggest an intervertebral disc extrusion at L3-4 that was confirmed at surgery.

A five-year-old female wirehaired dwarf dachshund was referred with a history of back pain of five days duration and paraparesis of 24 hours duration. On referral, the dog was ambulatory with severe ataxia and weakness in both pelvic limbs. Clinical signs were most pronounced on the right side. Spinal reflexes varied from normal to exaggerated, but postural reactions were absent in both pelvic limbs. Deep pain perception was normal and there were no signs of hyperaesthesia on palpation of the spine. The symptoms indicated a lesion localized between the T3 and L3 spinal cord segments. Radiographs of the thoracolumbar spine revealed an indistinct cloudy shadow within the intervertebral foramen of L3-4. Additionally, calcification at the intervertebral disc spaces T12-13, L3-4 and L7-S1 was identified. CT revealed a disc extrusion lateralised to the right, causing severe spinal cord compression at L3-4 (Figure 5) and moderate compression at L4-5.

A right hemilaminectomy was performed at L3 to L5 through a dorsal incision. Material mainly consisting of degenerated nucleus pulposus and fragments of annulus fibrosus was removed from the spinal canal. A slot was created in the lateral part of the L3-4 annulus and accessible intervertebral disc material was removed by curettage. A 2-3 mm thick layer of subcutaneous fat was placed over the hemilaminectomy site before closure.

**Figure 6:**
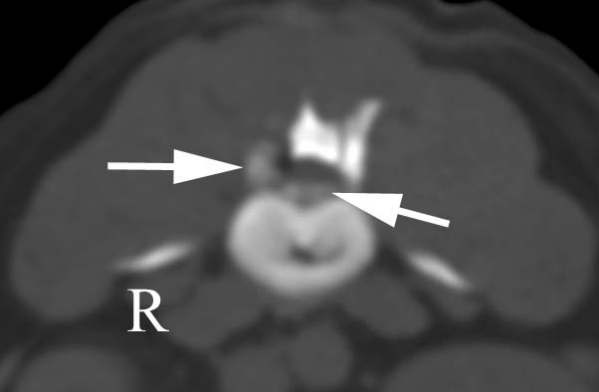
**CT scan in bone window through L3-4 four days after right sided hemilaminectomy**. The scan reveals calcified material (arrows) ventrally and laterally to the right of a compressed spinal cord. There is less calcification of the intervertebral disc compared to Figure 5. These findings suggest a recurrent disc extrusion that was confirmed at surgery.

For the first day following surgery, neurological function was similar compared with preoperative function. On the second day, signs of back pain were present and the weakness in both pelvic limbs was increased. The condition gradually deteriorated and on the fourth postoperative day, examination revealed a depressed mental status and a non-ambulatory paraparesis. Deep pain perception was normal. CT was repeated and revealed calcified disc material ventrally and to the right of a compressed spinal cord at L3-4 (Figure 6). A second surgery was performed and abundant disc material was found beneath the fat graft at L3-4. The disc material was removed and a new fat graft placed at the hemilaminectomy site. The wound was closed routinely. No more postoperative complications appeared and at examination three weeks following the second surgery the dog was ambulatory with very mild pelvic limb ataxia.

## Discussion

The three dogs presented in this report showed deterioration of clinical signs from two to seven days after hemilaminectomy. The deterioration was caused by recurrence of disc extrusion at the initial herniation site and shows that disc extrusions in chondrodystrophic dogs (mainly Hansen type I protrusions) may progress over several days and with a stepwise accumulation of disc material in the spinal canal.

A second spinal cord compression caused by more extruded disc material was found at the hemilaminectomy site in all the three dogs in this report. This finding shows that hemilaminectomy alone is not a surgical procedure that prevents a recurrence of extruded disc material from causing clinically significant spinal cord compression.

The influence of the autogeneous fat grafts on development of the second spinal cord compressions is uncertain. These grafts are detrimental if they make it difficult for the recurrent portion of extruded disc material to move spontaneously away from the spinal cord, nerve-roots and meninges. On the other hand, fat grafts are similar to epidural fat and could postoperatively prevent an unfavourable scar formation at the hemilaminectomy site [5]. Early recurrence of disc extrusion with compression of the spinal cord is also found in dogs subjected to hemilaminectomy without fat graft or other material at the hemilaminectomy site [3].

The term 'early recurrence' describes a complication to surgical management. If the three dogs in this report had been subjected to conservative treatment instead of decompressive surgery with removal of extruded disc material, they would have been expected to develop a more serious degree of spinal cord compression. In that case, a progressive development of a more serious neurological dysfunction and not a gradual improvement followed by worsening, would probably have characterized the clinical picture.

Proper fenestration of the herniated disc at the time of surgical decompression may counteract continued extrusion postoperatively. The benefit of fenestration should increase with increasing amounts of degenerated nucleus material remaining in the disc after the initial extrusion. Calcification at the intervertebral disc space, which is indicative of residual degenerated nucleus material, was seen at the herniated disc space on preoperative radiographs of all three dogs in this report. Findings that support the need for fenestration were therefore present and so this procedure could have also been performed on dogs 1 and 2 at the time of their initial surgeries.

In dog 3, the herniated disc was fenestrated at the time of hemilaminectomy. Nevertheless, recurrence of extrusion appeared at the same level within four days. This observation shows that fenestration through a dorsal approach with creation of a slot in the lateral part of the annulus is a procedure that does not completely prevent the disc from making a second extrusion. Dorsolateral and lateral approaches are also described for fenestration of thoracolumbar discs in dogs [6]. In a cadaveric study, Morelius *et al. *[7] found the lateral approach more efficient than both the dorsal and the dorsolateral approaches for the removal of nucleus material from thoracolumbar discs. Power-assisted fenestration is also reported to be more efficient than manual blade fenestration [8]. Nevertheless, Forterre *et al. *[3] found remaining disc material in all nine discs even after power-assisted fenestration following a lateral approach. It seems that complete removal of nucleus material cannot be expected from fenestration of thoracolumbar discs in dogs and the risk for a recurrent extrusion will therefore be present even after such a procedure.

In dog 3, nucleus material that remained in the L3-4 disc after fenestration soon extruded dorsally into the spinal canal. This occurrence showed that the path of least resistance for residual nucleus material was through the spontaneously formed dorsal rupture in the annulus and not through the lateral slot. Just making a slot without thoroughly removing residual nucleus material therefore seems insufficient to avoid early recurrence of disc extrusion.

The three dogs presented in this report showed postoperative signs of either no or temporary improvement followed by deterioration in neurological status. These signs were the main reasons for reexamination including CT. Temporary pain is another postoperative finding in dogs with early recurrence of disc extrusion [3]. In addition, some dogs display MRI-recurrence without clinical signs [3]. It is likely that some dogs may also show vague symptoms such as an absence of improvement or delay in improvement after surgery. On suspicion of recurrence of disc extrusion, re-examination including special imaging techniques should be done as soon as possible to make a reliable diagnosis for further treatment.

The second surgery that was performed on the three dogs in this report was a short and simple soft-tissue procedure that did not require a spinal bur or other special instruments. An evaluation of costs versus benefits will therefore justify a recommendation of re-operation as soon as possible after the diagnosis of an early recurrent disc extrusion is made.

## Conclusion

Thoracolumbar disc extrusions in chondrodystrophic dogs (Hansen type I protrusions) may progress over several days and are therefore not necessarily completed the day they are diagnosed. Further extrusion of disc material should be suspected particularly in cases that show an unexpected deterioration in neurological function within the first week after hemilaminectomy. The presence of calcified material at the herniated disc space is an indication to perform fenestration at the time of surgical decompression.

## Consent

Written informed consent was obtained from the owners for publication of this case report and accompanying images. A copy of the written consent is available for review by the Editor-in-Chief of this journal.

## Competing interests

The authors declare that they have no competing interests.

## Authors' contributions

ØS carried out the clinical examination of dog 2 and 3, performed the surgeries and is the main author of the paper. NO carried out the diagnostic imaging and KHJ did the clinical examination of dog 1. All authors read and approved the final manuscript.

## References

[B1] Fingeroth JM Fenestration: pros and cons Probl Vet Med 1989 1 445 466 2520126

[B2] Brisson BA Moffatt SL Swayne SL Parent JM Recurrence of thoracolumbar intervertebral disk extrusion in chondrodystrophic dogs after surgical decompression with or without prophylactic fenestration: 265 cases (1995-1999) J Am Vet Med Assoc 2004 224 1808 1814 10.2460/javma.2004.224.1808 15198267

[B3] Forterre F Konar M Spreng D Jaggy A Lang J Influence of intervertebral disc fenestration at the herniation site in association with hemilaminectomy on recurrence in chondrodystrophic dogs with thoracolumbar disc disease: a prospective MRI study Vet Surg 2008 37 399 405 10.1111/j.1532-950X.2008.00394.x 18564265

[B4] Stigen Ø Kolbjørnsen Ø Calcification of intervertebral discs in the dachshund: a radiographic and histopathologic study of 20 dogs Acta Vet Scand 2007 49 39 10.1186/1751-0147-49-39 18154641 PMC2262089

[B5] Sharp NJH Wheeler SJ Rodenhuis J Thoracolumbar disc disease Small Animal Spinal Disorders, Diagnosis and Surgery 2005 2 Edinburgh: Elsevier Mosby 121 159 full_text

[B6] Piermattei DL Johnson KA An Atlas of Surgical Approaches to the Bones and Joints of the Dog and Cat 2004 4 Philadelphia, WB Saunders 78 91

[B7] Morelius M Bergadano A Spreng D Schawalder P Doherr M Forterre F Influence of surgical approach on the efficacy of the intervertebral disk fenestration: a cadaveric study J Small Anim Pract 2007 48 87 92 10.1111/j.1748-5827.2007.00269.x 17286661

[B8] Holmberg DL Palmer NC VanPelt D Willan AR A comparison of manual and power-assisted thoracolumbar disk fenestration in dogs Vet Surg 1990 19 323 327 10.1111/j.1532-950X.1990.tb01199.x 2145687

